# Similar Inflammatory Biomarkers Reflect Different Platelet Reactivity in Percutaneous Coronary Intervention Patients Treated With Clopidogrel: A Large-Sample Study From China

**DOI:** 10.3389/fcvm.2021.736466

**Published:** 2021-10-04

**Authors:** Jiawen Li, Deshan Yuan, Lin Jiang, Xiaofang Tang, Jingjing Xu, Ying Song, Jue Chen, Shubin Qiao, Yuejin Yang, Runlin Gao, Bo Xu, Jinqing Yuan, Xueyan Zhao

**Affiliations:** State Key Laboratory of Cardiovascular Disease, Fuwai Hospital, National Center for Cardiovascular Diseases, Chinese Academy of Medical Sciences and Peking Union Medical College, Beijing, China

**Keywords:** clopidogrel, inflammation, platelet reactivity, coronary heart disease, thromboelastogram

## Abstract

**Background:** Platelet reactivity is closely associated with adverse events in percutaneous coronary intervention (PCI) patients. Inflammation plays a crucial role in the development of coronary heart disease (CHD).

**Aim:** To investigate the association of inflammatory biomarkers such as leukocyte count and high-sensitivity C reactive proteins (hs-CRP) with platelet reactivity in PCI patients treated with clopidogrel.

**Method:** We examined 10,724 consecutive PCI patients in Fuwai hospital from January 2013 to December 2013. High on-treatment platelet reactivity (HTPR) was defined as adenosine diphosphate (ADP)-induced platelet maximum amplitude [MA(ADP)] of thromboelastogram (TEG) > 47 mm, and low on-treatment platelet reactivity (LTPR) MA(ADP) < 31 mm.

**Results:** Finally, 6,772 PCI patients treated with clopidogrel who had the results of postoperative TEG were enrolled. Among them, 2,070 (30.57%) presented HTPR and 2,568 (37.92%) presented LTPR. As for LTPR, multivariate logistic regression showed that leukocyte count (OR: 1.153, 95% CI 1.117–1.191) and hs-CRP (OR: 0.920, 95% CI 0.905–0.936) were independent predictors, along with diabetes mellites, hemoglobin, platelet count and glucose. As for HTPR, multivariate logistic regression showed that leukocyte count (OR: 0.885, 95% CI 0.854–0.917) and hs-CRP (OR: 1.094, 95% CI 1.077–1.112) were independent predictors, along with sex, hemoglobin, platelet count and glucose.

**Conclusions:** This was the first large real-world study reporting that both leukocyte count and hs-CRP were the independent factors for platelet reactivity in PCI populations treated with clopidogrel, among which higher leukocyte count was associated with more LTPR while higher hs-CRP was associated with more HTPR, providing new insights on individualized antiplatelet therapy.

## Introduction

Platelet reactivity varies in percutaneous coronary intervention (PCI) patients treated with clopidogrel (1), and platelet participates in thrombosis and bleeding events. Previous studies have established in patients with coronary heart disease (CHD) that high on-treatment platelet reactivity (HTPR) is an independent risk factor for thrombotic events (2–4), while low on-treatment platelet reactivity (LTPR) is an independent risk factor for bleeding events (5–8). Therefore, platelet reactivity controlled within normal range might contribute to the reduction of thrombosis and bleeding events in patients with CHD. It's reported that many factors have an impact on individual platelet reactivity, including clinical characteristics, inheritance, drug interaction and so on (9). Hence, it is crucial to deeply explore relevant factors affecting platelet reactivity in PCI populations.

In recent years, inflammation has been confirmed to be closely associated with the occurrence and development of CHD, recieving increasing attention (10, 11). As inflammatory biomarkers, leukocyte count increases rapidly in response to acute inflammation and high-sensitivity C reactive protein (hs-CRP) is an acute phase reactant indicating the presence of active inflammation. Inflammatory biomarkers have also become the therapeutic target for CHD in era of “poststatins,” as recent CANTOS (12) and LoDoCo2 (13) trails established that anti-inflammation improves the prognosis of CHD. Previous studies have shown that elevated leukocyte count (14–17) and hs-CRP (18–20) were associated with bleeding and thrombotic adverse events in PCI patients, however, the mechanism of the increased risk is unknown.

At present, there are less studies conducted between inflammatory biomarkers and clopidogrel-related platelet reactivity, and beyond their inconsistent conclusions, there is also a lack of large sample (18, 21–23). Whether inflammation affects platelet reactivity needs further study. This study aimed to investigate the effect of inflammatory markers such as leukocyte count and hs-CRP on platelet reactivity in a large PCI population (*n* = 6,772) from China, which may be helpful to provide reference for the potential mechanism and clinical decision making.

## Methods

### Study Population

**Figure 1:**
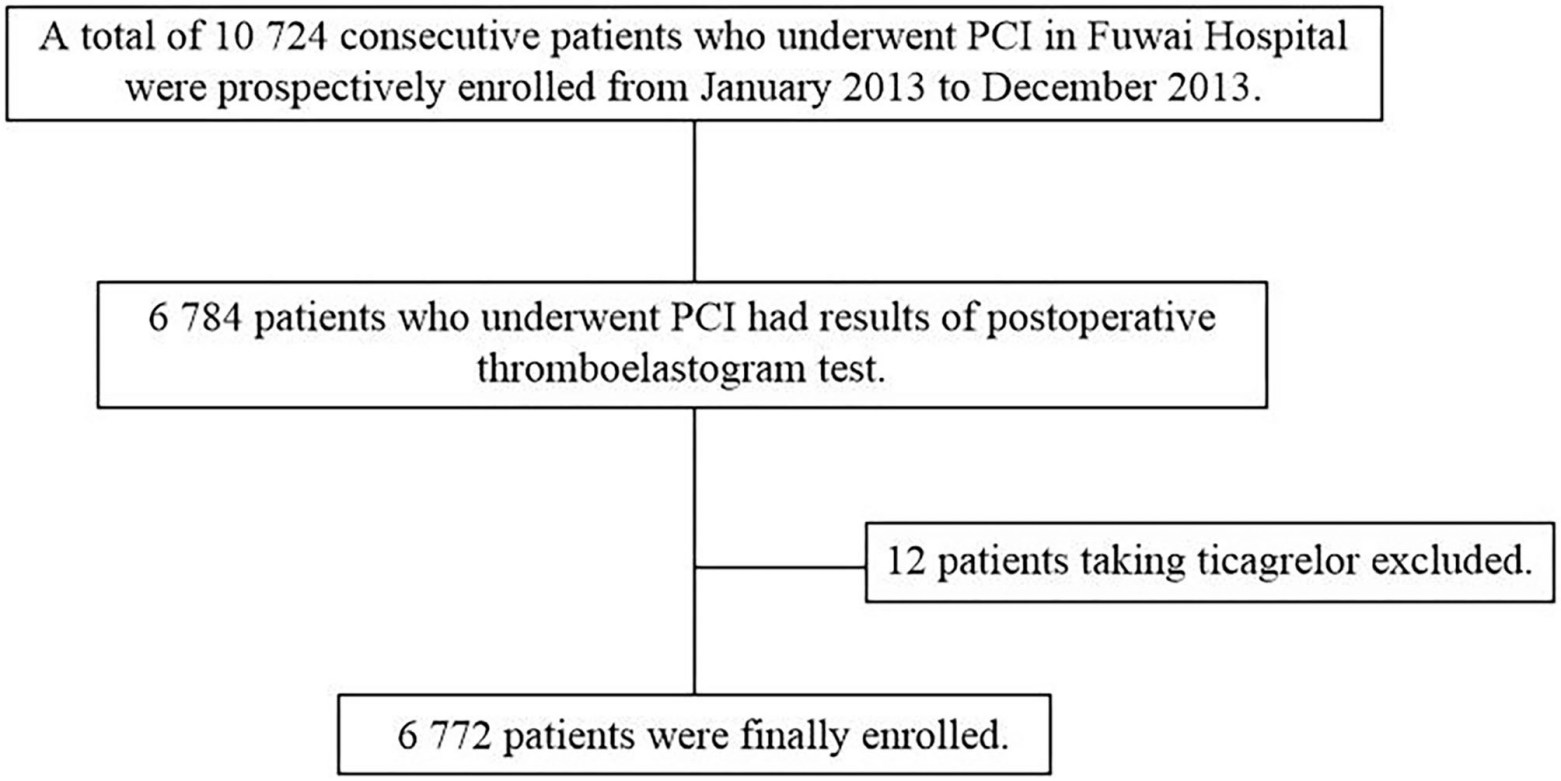
Flowchart of the study. PCI, percutaneous coronary intervention.

This prospective, single-center, observational study consecutively enrolled 10,724 PCI patients with CHD who treated with dual antiplatelet therapy in Fuwai hospital from January 2013 to December 2013, among whom 6,784 had the results of postoperative thromboelastogram (TEG) in the real world, and 12 patients treated with ticagrelor were excluding. Finally, a total of 6,772 patients were included in this study (Figure 1).

All subjects received asprin and clopidogrel before the procedure. Those who had not taken antiplatelet drugs before procedure were given 300 mg asprin and 300–600 mg clopidogrel for loading doses. After the procedure, all subjects were given aspirin 100 mg per day and clopidogrel 75 mg per day for at least 1 year.

The study protocol was approved by the Fuwai hospital institutional review board, and before the study all patients were provided written informed consent.

### Determination of Inflammatory Biomarkers

Blood sample was collected in all patients after fasting in the morning within 24 h after admission. The inflammatory biomarkers in our study consisted of leukocyte count and hs-CRP. Using an automated biochemical analyzer to determine biochemical indicators (LABOSPECT 008, HITACHI, Japan), including hs-CPR, low-density lipoprotein-cholesterol (LDL-C), high-density lipoprotein cholesterol (HDL-C), total cholesterol (TC), triglyceride, glucose and estimated glomerular filtration rate (eGFR), etc. Using a Sysmex XN 2000 automated blood cell counter (Sysmex Corporation, Kobe, Japan) to determine leukocyte count, hemoglobin, platelet count and mean platelet volume.

### Determination and Definition of Platelet Reactivity

This study adopted the adenosine diphosphate (ADP)-induced platelet maximum amplitude [MA(ADP)] of TEG to evaluate platelet reactivity, which reflected the maximum amplitude of clots after using P_2_Y_12_ receptor antagonists. In the next morning after PCI, patients were taken venous blood sample for point of care TEG on the supine position. The parameters of TEG were measured by TEG@5,000 thromboelastograph hemostasis system (American Haemoscope Corporation). According to the consensus of definition of platelet reactivity (24), HTPR was MA(ADP) >47 mm, normal on-treatment platelet reactivity (NTPR) was MA(ADP) 31–47 mm, and LTPR was MA(ADP) <31 mm in this study.

### Statistical Analysis

Continuous variables were expressed as mean ± standard deviation in nomal distribution and median (interquartile range) in non-nomal distribution. Categorical variables were expressed as numbers (percentages). Analysis of variance or Kruskal-Wallis *H*-test was used to compare continuous variables, while Chi-square test or Fisher's exact test was applied to compare categorical variables between the three groups. Pearson correlation was used to assess the correlation between leukocyte count and hs-CRP. Leukocyte count and hs-CRP were modeled as continuous variables in our study. Univariate logistic regression was used to analyse the related factors of HTPR or LTPR. Enrolling the siginicant variables from univariate logistic regression, multivariate logistic regression analysis was conducted to evaluate the association of inflammatory markers (leukocyte count and hs-CRP) with HTPR and LTPR. The effect of leukocyte count on LTPR and the effect of hs-CRP on HTPR in different subgroups were assessed by multivariate logistic regression models with tests for interaction. The propensity score matching (PSM) was performed to make a sensitivity analysis. We matched the significant variables according the univariate logistic regression analysis in a 1:1 nearest neighbor manner with a caliper width equal to 0.02. Statistical significance was considered as two-sided *P*-value of <0.05. All analyses were performed using SPSS software version 22.0 (IBM Corp., Armonk, New York, USA).

## Results

### Baseline Characteristics

A total of 6,772 consecutive patients were enrolled in the present study. The average age was 58.23 ± 10.28 years, 5,252 (77.6%) were male, 3,877 (57.3%) presented with acute coronary syndrome (ACS) and 10 (0.15%) were on anticoagulant treatment. The average MA(ADP) of TEG was 35.82 ± 17.62 mm. Patients were divided into three groups according to the value of MA(ADP): LTPR (2,568, 37.92%), NTPR (2,134, 31.51%) and HTPR (2,070, 30.57%). Comparison was performed among the three groups: sex, age, MA(ADP), leukocyte count, hs-CRP, smoking history, ACS, hypertension, diabetes mellitus, prior myocardial infarction, prior PCI, hemoglobin, platelet count, LDL-C, TC, glucose and eGFR were significantly different (*P* all <0.05). There was no difference in other indicators (*P* all >0.05) (Table 1).

**Table 1:** Baseline characteristics of patients according to platelet reactivity.

**Parameter**	**LTPR**	**NTPR**	**HTPR**	***p*** **-value**
	**(*N* = 2,568)**	**(*N* = 2,134)**	**(*N* = 2,070)**	
Sex [male, *n* (%)]	2,168 (84.40)	1,781 (83.50)	1,303 (62.90)	<0.001
Age, year	57.080 ± 10.088	57.958 ± 10.422	59.950 ± 10.157	<0.001
BMI, kg/m^2^	25.947 ± 3.208	26.007 ± 3.113	25.968 ± 3.165	0.808
MA(ADP), mm	16.689 ± 8.822	39.621 ± 4.551	55.622 ± 6.085	<0.001
Leukocyte count, 10^9^/L	6.999 ± 1.937	6.689 ± 1.784	6.707 ± 1.927	<0.001
Hs-CRP, mg/L	1.410 (2.020)	1.580 (2.480)	2.095 (5.103)	<0.001
Smoking history, *n* (%)	1,612 (62.80)	1,309 (61.30)	1,013 (48.90)	<0.001
ACS, n (%)	1,432 (55.80)	1,203 (56.40)	1,242 (60.00)	0.009
Hyperlipidemia, *n* (%)	1,755 (68.30)	1,459 (68.40)	1,402 (67.70)	0.879
Hypertension, *n* (%)	1,633 (63.60)	1,378 (64.60)	1,388 (67.10)	0.044
Diabetes Mellitus, *n* (%)	711 (27.70)	647 (30.30)	698 (33.70)	<0.001
COPD, *n* (%)	56 (2.20)	48 (2.20)	49 (2.40)	0.913
Family history of CHD, *n* (%)	640 (24.90)	529 (24.80)	501 (24.20)	0.830
Cerebrovascular disease history, *n* (%)	238 (9.30)	235 (11.00)	232 (11.20)	0.054
Peripheral vascular disease, *n* (%)	71 (2.80)	54 (2.50)	69 (3.30)	0.275
Prior myocardial infarction, *n* (%)	536 (20.90)	445 (20.90)	356 (17.20)	0.002
Prior PCI, *n* (%)	674 (26.20)	527 (24.70)	461 (22.30)	0.007
Prior CABG, *n* (%)	104 (4.00)	99 (4.60)	74 (3.60)	0.217
LVEF, %	63.189 ± 7.034	62.754 ± 7.217	62.777 ± 7.314	0.062
Hemoglobin, g/L	146.596 ± 14.532	143.808 ± 14.344	136.789 ± 14.979	<0.001
PLT, 10^9^/L	201.158 ± 53.475	198.773 ± 51.238	216.628 ± 58.404	<0.001
MPV, fL	10.613 ± 0.914	10.599 ± 0.922	10.586 ± 0.901	0.589
LDL-C, mmol/L	2.414 ± 0.875	2.465 ± 0.902	2.592 ± 0.903	<0.001
HDL-C, mmol/L	1.027 ± 0.269	1.020 ± 0.267	1.036 ± 0.274	0.175
TC, mmol/L	4.104 ± 1.039	4.155 ± 1.071	4.328 ± 1.080	<0.001
Triglyceride, mmol/L	1.771 ± 1.059	1.754 ± 1.079	1.799 ± 0.996	0.374
Glucose, mmol/L	5.984 ± 1.823	6.043 ± 1.892	6.263 ± 2.119	<0.001
eGFR, ml/min	92.659 ± 14.265	92.150 ± 14.496	89.789 ± 15.479	<0.001

*BMI, body mass index; MA(ADP), adenosine diphosphate (ADP)-induced platelet maximum amplitude; Hs-CRP, high-sensitivity C reactive protein; ACS, acute coronary syndrome; COPD, chronic obstructive pulmonary disease; CHD, coronary heart disease; PCI, percutaneous coronary intervention; CABG, coronary artery bypass graft; LVEF, left ventricle ejection fraction; PLT, platelet count; MPV, mean platelet volume; LDL-C, low-density lipoprotein cholesterol; HDL-C, high-density lipoprotein cholesterol; TC, total cholesterol; eGFR, estimated glomerular filtration rate*.

### Leukocyte Count and hs-CRP in Different Groups

The mean of leukocyte count in the group of LTPR, NTPR and HTPR were 7.00 ± 1.94, 6.69 ± 1.78 and 6.71 ± 1.93 10^9^/L respectively which were significantly different (*P* < 0.001). Compared with that of LTPR group, the leukocyte count of NTPR group (*P* < 0.001) and HTPR group (*P* < 0.001) decreased significantly. No significant difference can be seen in the leukocyte count between HTPR group and NTPR group (*P* = 1.000).

The median (interquartile range) of hs-CRP in the group of LTPR, NTPR, and HTPR were 1.41 (2.02), 1.58 (2.48) and 2.06 (5.10) mg/L respectively which were significantly different (*P* < 0.001). Compared with that of HTPR group, the hs-CRP levels of LTPR group (*P* < 0.001) and NTPR group (*P* < 0.001) decreased significantly. Compared with that of NTPR group, the hs-CRP levels of LTPR group reduced significantly (*P* < 0.001).

The leukocyte count showed significant correlation with hs-CRP (*r* = 0.329, *P* < 0.001).

### Multivariate Logistic Analysis for Platelet Reactivity

When LTPR was the dependent variable in a multivariate logistic regression model, the independent variables were leukocyte count, hs-CRP and the significant factors in univariate analysis (sex, age, smoking history, diabetes mellitus, cerebrovascular disease history, prior PCI, left ventricle ejection fraction, hemoglobin, platelet count, LDL-C, TC, glucose and eGFR). Multivariate logistic regression analysis showed that leukocyte count (adjusted OR 1.153, 95% CI 1.117–1.191, *P* < 0.001) and hs-CRP (adjusted OR 0.920, 95% CI 0.905–0.936, *P* < 0.001) were independently associated with LTPR (see Table 2). Besides, diabetes mellitus (adjusted OR 0.868, 95% CI 0.759–0.993), hemoglobin (adjusted OR 1.023, 95% CI 1.019–1.028), platelet count (adjusted OR 0.997, 95% CI 0.996–0.998), and glucose (adjusted OR 0.962, 95% CI 0.931–0.994) were also independently associated with LTPR (see Table 2).

**Table 2:** Logistic regression for LTPR.

**Parameter**	**Univariate logistic regression**			**Multivariate logistic regression**		
	**Crude OR**	**95% CI**	***p*** **-value**	**Adjusted OR**	**95% CI**	***p*** **-value**
Sex	1.968	1.734–2.234	<0.001	1.095	0.923–1.299	0.297
Age	0.983	0.978–0.987	<0.001	0.995	0.988–1.001	0.127
BMI	0.996	0.981–1.011	0.604	–	–	–
Leukocyte count	1.087	1.059–1.115	<0.001	1.153	1.117–1.191	<0.001
Hs-CRP	0.926	0.912–0.940	<0.001	0.920	0.905–0.936	<0.001
Smoking history	1.367	1.236–1.511	<0.001	1.010	0.895–1.140	0.868
ACS	0.907	0.821–1.001	0.053	–	–	–
Hyperlipidemia	1.013	0.912–1.126	0.806	–	–	–
Hypertension	0.908	0.819–1.006	0.065	–	–	–
Diabetes mellitus	0.814	0.731–0.907	<0.001	0.868	0.759–0.993	0.039
COPD	0.944	0.677–1.316	0.734	–	–	–
Family history of CHD	1.024	0.914–1.148	0.680	–	–	–
Cerebrovascular disease history	0.817	0.693–0.963	0.016	0.907	0.763–1.078	0.268
Peripheral vascular disease	0.943	0.701–1.269	0.700	–	–	–
Prior myocardial infarction	1.121	0.992–1.267	0.068	–	–	–
Prior PCI	1.158	1.034–1.297	0.011	1.099	0.975–1.239	0.120
Prior CABG	0.983	0.767–1.261	0.895	–	–	–
LVEF	1.008	1.001–1.015	0.019	1.007	1.000–1.015	0.057
Hemoglobin	1.029	1.025–1.033	<0.001	1.023	1.019–1.028	<0.001
PLT	0.998	0.997–0.999	<0.001	0.997	0.996–0.998	<0.001
MPV	1.025	0.972–1.082	0.360	–	–	–
LDL-C	0.865	0.817–0.915	<0.001	0.952	0.810–1.120	0.553
HDL-C	0.985	0.821–1.182	0.869	–	–	–
TC	0.885	0.845–0.928	<0.001	0.916	0.798–1.051	0.212
Triglyceride	0.996	0.950–1.044	0.867	–	–	–
Glucose	0.955	0.931–0.981	0.001	0.962	0.931–0.994	0.021
eGFR	1.008	1.004–1.011	<0.001	1.000	0.995–1.004	0.942

*BMI, body mass index; MA(ADP), adenosine diphosphate (ADP)-induced platelet maximum amplitude; WBC, white blood cell; Hs-CRP, high-sensitivity C reactive protein; ACS, acute coronary syndrome; COPD, chronic obstructive pulmonary disease; CHD, coronary heart disease; PCI, percutaneous coronary intervention; CABG, coronary artery bypass graft; LVEF, left ventricle ejection fraction; PLT, platelet count; MPV, mean platelet volume; LDL-C, low-density lipoprotein cholesterol; HDL-C, high-density lipoprotein cholesterol; TC, total cholesterol; eGFR, estimated glomerular filtration rate*.

When HTPR was the dependent variable in a multivariate logistic regression model, the independent variables were leukocyte count, hs-CRP and the significant factors in univariate analysis (sex, age, smoking history, ACS, hypertension, diabetes mellitus, prior myocardial infarction, prior PCI, hemoglobin, platelet count, LDL-C, TC, glucose and eGFR). Multivariate logistic regression analysis showed that leukocyte count (adjusted OR 0.885, 95% CI 0.854–0.917, *P* < 0.001) and hs-CRP(adjusted OR 1.094, 95% CI 1.077–1.112, *P* < 0.001) were independently associated with HTPR (see Table 3). Besides, sex (adjusted OR 0.636, 95% CI 0.537–0.754), hemoglobin (adjusted OR 0.972, 95% CI 0.968–0.977), platelet count (adjusted OR 1.005, 95% CI 1.004–1.007), and glucose (adjusted OR 1.056, 95% CI 1.020–1.092) were also independently associated with HTPR (see Table 3).

**Table 3:** Logistic regression for HTPR.

**Parameter**	**Univariate logistic regression**			**Multivariate logistic regression**		
	**Crude OR**	**95% CI**	***p*** **-value**	**Adjusted OR**	**95% CI**	***p*** **-value**
Sex	0.324	0.288–0.365	<0.001	0.636	0.537–0.754	<0.001
Age	1.024	1.019–1.029	<0.001	1.006	0.998–1.013	0.123
BMI	0.999	0.983–1.016	0.944	–	–	–
Leukocyte count	0.958	0.931–0.985	0.002	0.885	0.854–0.917	<0.001
Hs-CRP	1.093	1.079–1.108	<0.001	1.094	1.077–1.112	<0.001
Smoking history	0.584	0.526–0.649	<0.001	0.995	0.870–1.138	0.942
ACS	1.177	1.059–1.307	0.002	1.027	0.915–1.154	0.649
Hyperlipidemia	0.972	0.870–1.086	0.611	–	–	–
Hypertension	1.143	1.025–1.275	0.017	0.953	0.846–1.074	0.429
Diabetes mellitus	1.253	1.121–1.400	<0.001	1.144	0.991–1.320	0.065
COPD	1.072	0.760–1.511	0.692	–	–	–
Family history of CHD	0.964	0.855–1.087	0.550	–	–	–
Cerebrovascular disease history	1.129	0.956–1.333	0.154	–	–	–
Peripheral vascular disease	1.263	0.937–1.702	0.126	–	–	–
Prior myocardial infarction	0.788	0.689–0.901	<0.001	1.004	0.861–1.169	0.964
Prior PCI	0.835	0.739–0.944	0.004	0.931	0.810–1.069	0.312
Prior CABG	0.822	0.626–1.078	0.156	–	–	–
LVEF	0.996	0.989–1.003	0.257	–	–	–
Hemoglobin	0.962	0.958–0.965	<0.001	0.972	0.968–0.977	<0.001
PLT	1.005	1.004–1.006	<0.001	1.005	1.004–1.007	<0.001
MPV	0.975	0.921–1.032	0.380	–	–	–
LDL-C	1.208	1.142–1.279	<0.001	1.013	0.851–1.205	0.888
HDL-C	1.176	0.973–1.423	0.094	–	–	–
TC	1.189	1.134–1.247	<0.001	1.140	0.982–1.324	0.085
Triglyceride	1.032	0.983–1.083	0.202	–	–	–
Glucose	1.066	1.039–1.093	<0.001	1.056	1.020–1.092	0.002
eGFR	0.988	0.985–0.992	<0.001	0.998	0.993–1.003	0.437

*BMI, body mass index; MA(ADP), adenosine diphosphate (ADP)-induced platelet maximum amplitude; WBC, white blood cell; Hs-CRP, high-sensitivity C reactive protein; ACS, acute coronary syndrome; COPD, chronic obstructive pulmonary disease; CHD, coronary heart disease; PCI, percutaneous coronary intervention; CABG, coronary artery bypass graft; LVEF, left ventricle ejection fraction; PLT, platelet count; MPV, mean platelet volume; LDL-C, low-density lipoprotein cholesterol; HDL-C, high-density lipoprotein cholesterol; TC, total cholesterol; eGFR, estimated glomerular filtration rate*.

### The Optimal Cutoff Point of Leukocyte Count and hs-CRP for Predicting Platelet Reactivity

**Figure 2:**
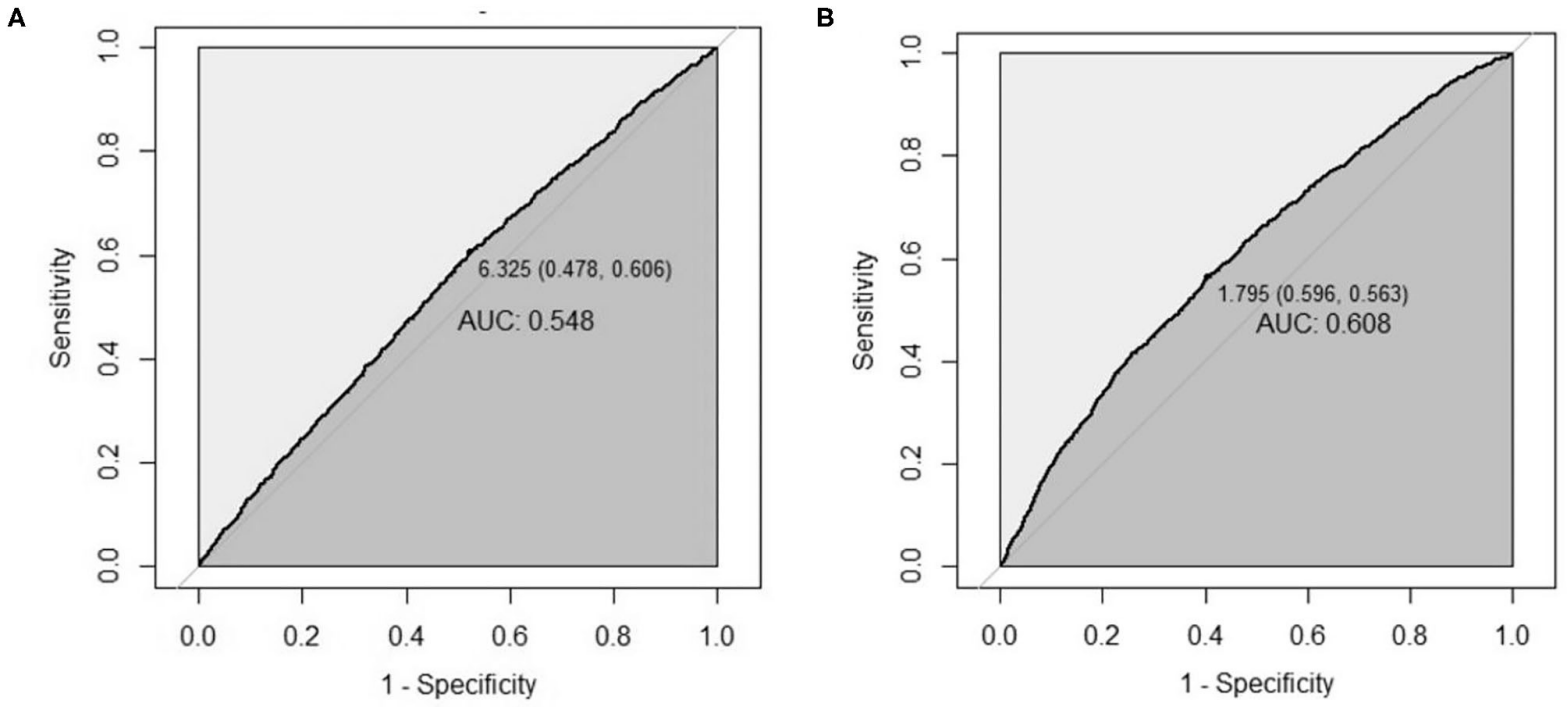
The AUC of leukocyte count and hs-CRP for predicting platelet reactivity. **(A,B)** The AUC of leukocyte count for predicting LTPR **(A)**, and the AUC of hs-CRP for predicting HTPR **(B)**. AUC, area under curve; LTPR, low on-treatment platelet reactivity; HTPR, high on-treatment platelet reactivity.

The area under curve (AUC) of leukocyte count predicting LTPR was 0.548 (95% CI: 0.534–0.562, *P* < 0.001) with 6.325 10^9^/L as the optimal cutoff point (Figure 2).

The AUC of hs-CRP predicting HTPR was 0.608 (95% CI: 0.593–0.622, *P* < 0.001) with 1.795 mg/L as the optimal cutoff point (Figure 2).

### Subgroup Analysis

**Figure 3:**
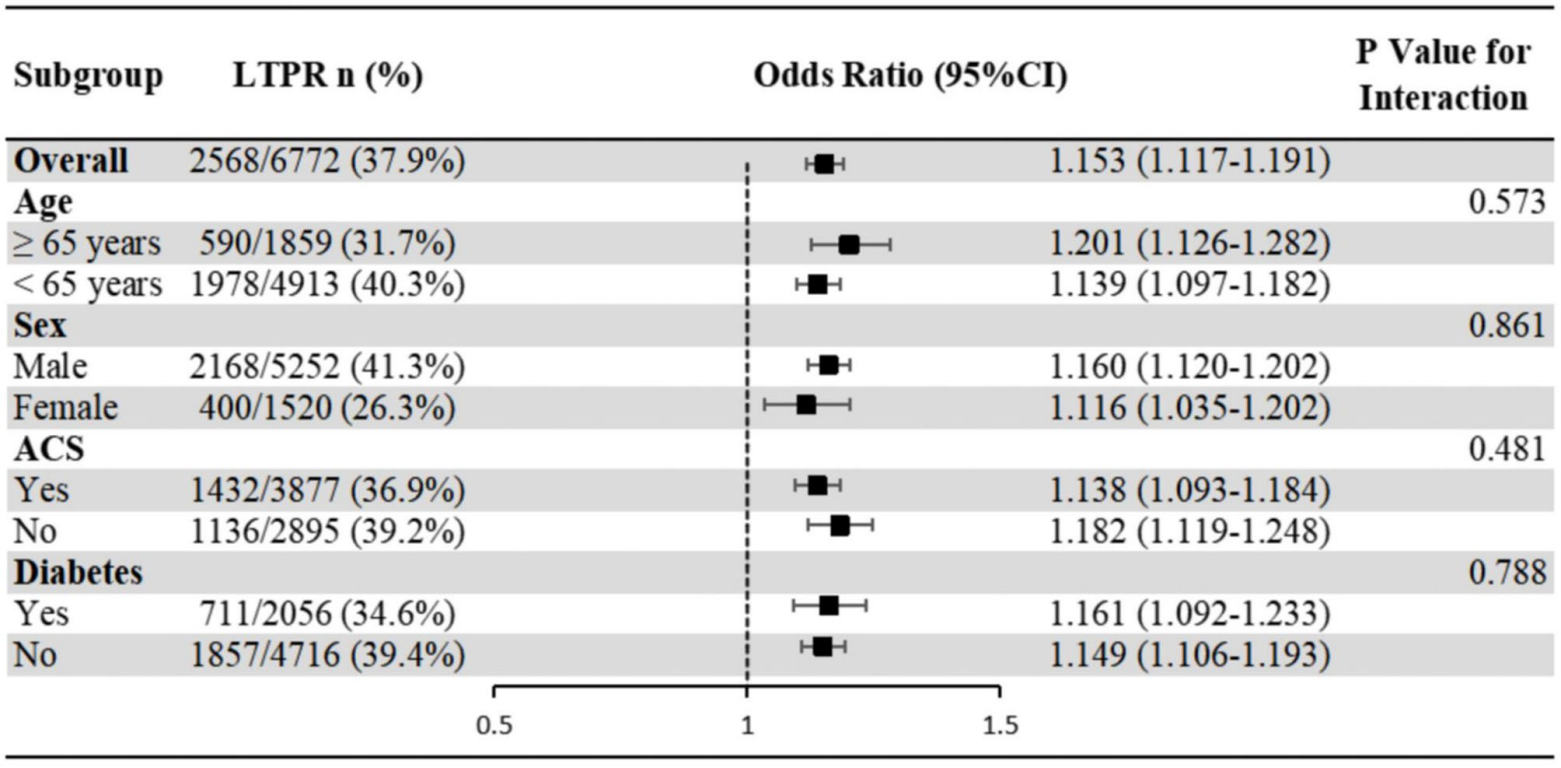
Subgroup analyses of the association of leukocyte count with LTPR. Odds ratios and 95% confidence intervals are shown for LTPR. *P*-value represents interaction test between the variable and the leukocyte count. ACS, acute coronary syndrome; LTPR, low on-treatment platelet reactivity.

The association of leukocyte count with LTPR showed no significant interaction with age, sex, ACS and diabetes populations (*P*-value for interaction >0.05) (Figure 3).

**Figure 4:**
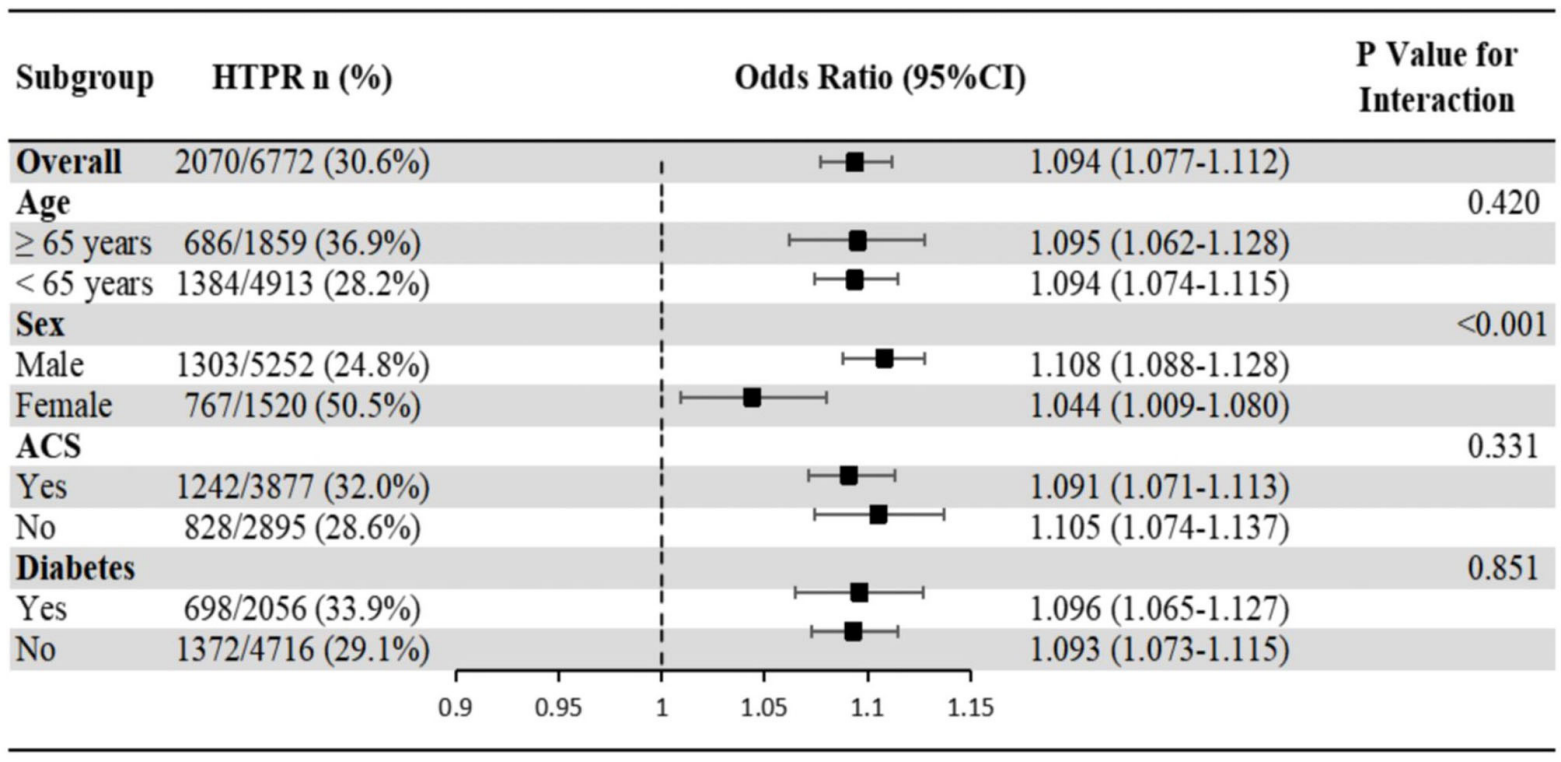
Subgroup analyses of the association of hs-CRP with HTPR. Odds ratios and 95% confidence intervals are shown for HTPR. *P*-value represents interaction test between the variable and hs-CRP. ACS, acute coronary syndrome; HTPR, high on-treatment platelet reactivity; hs-CRP, high-sensitivity C reactive protein.

The association of hs-CRP with HTPR showed no significant interaction with age, ACS and diabetes populations (*P*-value for interaction >0.05), but significant interaction with sex (*P*-value for interaction <0.001), which showed that the association of hs-CRP with HTPR in male was more significant than that in female (Figure 4).

### Sensitivity Analysis

After PSM, we got LTPR (*n* = 2,402) and non-LTPR (*n* = 2,402) and found that univariable and multivariable analyses showed that higher leukocyte count level was strongly associated with increased risks of LTPR (adjusted OR 1.128, 95% CI 1.091–1.166; *P*< 0.001) (Supplementary Table 1).

After PSM, we got HTPR (*n* = 1,894) and non-HTPR (*n* = 1,894) and found that univariable and multivariable analyses showed that higher hs-CRP level was strongly associated with increased risks of HTPR (adjusted OR 1.091, 95% CI 1.071–1.111; *P* < 0.001) (Supplementary Table 2).

## Discussion

In this large sample (*n* = 6,772) and real-world study, we evaluated the association of inflammatory biomarkers with platelet reactivity. Our major findings in the present study were as follows: (1) Leukocyte count and hs-CRP were independently associated with platelet reactivity, among which elevated leukocyte count was related to more LTPR while elevated hs-CRP was related to more HTPR. (2) Diabetes mellitus, hemoglobin, platelet count and glucose were independent factors of LTPR, and sex, hemoglobin, platelet count and glucose were independent factors of HTPR.

It has been commonly acknowledged that inflammatory response triggers changes in blood inflammatory markers. Both leukocyte count and hs-CRP are inflammatory biomarkers and show positively correlated; our results showed that leukocyte count and hs-CRP were independently related to platelet reactivity, but interestingly, their effects were entirely distinguished from each other: the higher leukocyte count was, the more likely the patient presented to LTPR while the higher hs-CRP, was the more likely the patient presented HTPR. Patients undergoing PCI treated with clopidogrel present great diversity in platelet reactivity (1). Previous studies showed that the incidence of HTPR was approximately 34.50–40.40% (2, 25, 26), while that of LTPR was about 11.20–19.00% (8, 25). Common with the previous studies partially, our study displayed that the incidence of HTPR was 30.57% in PCI populations, but that of LTPR was 37.92%, suggesting that the incidence of LTPR in Chinese PCI population was higher, which may also explain different bleeding risks between European, American and Asian, since the bleeding risk of Asian is generally thought to be higher when receiving the same anti-platelet therapy (27). Now there are many methods to detect platelet function, including light transmission aggregometry, TEG, multiple electrode aggregometry (MEA), vasodilator stimulated phosphoprotein phosphorylation (VASP) and VerifyNow. To the best of our knowledge, the present study was the first to investigate the association between inflammation and platelet reactivity defined by MA(ADP) of TEG. As a result, inflammatory biomarkers, namely leukocyte count and hs-CRP, were independent factors of platelet reactivity.

Leukocytes are the earliest recognized inflammatory marker. Previous studies have shown that the higher leukocyte was, the higher death risk and the worse prognosis presented in patients with CHD (14, 15). Besides, leukocytes are also considered to be an independent risk factor for bleeding in patients with CHD. Leukocyte count was listed as an important variable in the traditional ACUITY (16) score for the evaluation of bleeding risk in ACS patients as well as in the PRECISE-DAPT score (17) recommended in recent guidelines. Further, Mehran et al. (28) established a risk score consisting of seven variables including leukocyte count, suggesting good predictive value for the bleeding risk in PCI patients. However, there was no explanation why the elevated leukocyte count increased bleeding risk and no relevant mechanisms reported. Against this background, our study reported for the first time that leukocyte count was an independent risk factor of LTPR, and the higher leukocyte count was, the more likely the patient appeared LTPR. As mentioned above, LTPR was associated with an increased risk of bleeding in the PCI population (5–8). Thereby, our opinion that high leukocyte count may increase the risk of bleeding by virtue of LTPR may provide new insights into the mechanisms. Athough a few previous studies based on relatively small samples have also explored the association between leukocyte and platelet reactivity, their results were inconsistent. Osmancik et al. (21) reported that the higher leukocyte count was, the less likely it was to appear HTPR tested by VerifyNow (PRU > 240) in 378 PCI patients, which was consistent with our finding. However, Bernlochner et al. (23) reported that leukocyte count >10 × 10^9^/L was independently associated with HTPR tested by MEA (ADP-induced platelet aggregation >468 AU^*^min) in 1,233 patients with stable CHD and Morel et al. (29) reported that leukocyte count >10,000/mm^3^ was independently associated with HTPR tested by VASP (PRI ≥ 61%) in 160 ACS patients with DM. The different platelet function testings and different CHD populations types may account for the differences from our results. A prior study showed that the results from different platelet function testings in the same population were less consistent (30). Including 6,772 PCI patients, our research has been the largest study so far and showed that in the real world, the higher leukocyte count was, the greater it was the likelihood to reflect LTPR defined by MA(ADP) of TEG, which suggested that we may be able to identify the high risk of bleeding by means of paying attention to patients with high leukocyte count after PCI.

As an indicator of inflammation, hs-CRP has been thought to be closely related to the risk of death and ischemic events in patients with CHD (18–20). The results of this study showed that the higher hs-CRP was, the patient presented more HTPR. Several previous studies demonstrated that clopidogrel-related HTPR was associated with increased risk of thrombotic events in patients with CHD after PCI (2–4). However, there are few studies on the association between hs-CRP and HTPR, and some smaller studies indicated that there was correlation between them. Caruso et al. (31) enrolled 29 ACS patients and reported that HTPR patients were in a prolonged proinflammatory environment. Jiang et al. (22) enrolled 203 STEMI patients and discovered that taking clopidogrel at moderate-high hs-CRP levels enhanced platelet aggregation in PCI patients. Adatia et al. (18) reported there was a correlation between hs-CRP and platelet reactivity (*r* = 0.14, *P* = 0.003), which enrolled 541 STEMI patients. Consistent with these smaller studies, we found that the higher hs-CRP was, the greater it was the likelihood to reflect HTPR defined by MA(ADP) of TEG. These suggested us to identify the high risk of thrombotic events, and patients with elevated hs-CRP might be able to choose more potent antiplatelet drugs, such as higher doses of clopidogrel or new antiplatelet drugs. Patti et al. (32) reported that the 150-mg/day clopidogrel maintenance dose was associated with stronger platelet inhibition and reduction of inflammation, compared with the currently recommended 75-mg/day regimen. Huang et al. (33) revealed a novel pharmacological function of ticagrelor in addition to its classic antiplatelet properties, which suggested that ticagrelor may serve as a potential therapeutic agent in NLRP3-associated diseases. Schnorbus et al. (34) found that prasugrel could better lower interleukin-6 and had endothelial protection compared with ticagrelor. The above studies suggested that we might pay attention to the ischemia risk in those with elevated hs-CRP and may be able to prescribe more active antiplatelet drugs in the future.

We also found that diabetes mellitus, hemoglobin levels, platelet count and glucose were also associated with platelet reactivity which was consistent with the previous studies (35–39). Even though we made these variables enter multivariate regression model as much as possible, inflammatory markers including leukocyte count and hs-CRP were still independent indicators of platelet reactivity, which implied that it's inflammation itself that strongly affected platelet reactivity in PCI patients.

In terms of platelet reactivity, the present study has been the largest real-world study so far and the first to investigate the association between inflammation and platelet reactivity using MA(ADP) of TEG as the indicator. Although it's established that higher leukocyte count increased bleeding risk and higher hs-CRP increased thrombosis risk, to our knowledge we did not see any discussions about the concrete mechanism, and our study confirmed that both leukocyte count and hs-CRP were independently associated with platelet reactivity, but their effects were totally different, which may explain the mechanism in part. The mechanism of different effects of two similar inflammatory markers on platelet reactivity needs further study. It is worth mentioning that subgroup analyses (age, sex, ACS and diabetes populations) and sensitivity analysis showed good reliability and stability in our results. The important findings appealed physicians to focus on leukocyte count and hs-CRP respectively in CHD patients with increased inflammation in the future to help identify the risk of bleeding and thrombosis, and then prescribed individual antiplatelet therapy.

However, the study also had some limitations. Firstly, the study was conducted in a single center, limiting the universality. Secondly, we did not record the proportion of chronic clopidogrel treatment and loading-dose treatment, so the duration of clopidogrel treatment maybe various individually in this study, which may bring about the diversity of platelet reactivity (40). Thirdly, HTPR and LTPR tested by different methods may lead to the diversity of the incidence of platelet reactivity. Fourthly, the predictive value of leukocyte count or hs-CRP to platelet reactivity is significant but small, according to the relatively low AUC values (0.608 and 0.548). Finally, although relevant variables were included for adjustment as more as possible in this study, there were still some related factors neglected, such as inheritance, which may have a certain impact on the results.

## Conclusion

The present study firstly reported that inflammatory biomarkers were the independent factors for platelet reactivity in a large real-world PCI populations treated with clopidogrel, among which higher leukocyte count was associated with more LTPR while higher hs-CRP was associated with more HTPR. Our findings might shed light on the mechanism of higher leukocyte count increased the risk of bleeding and higher hs-CRP increased the risk of thrombosis. In future, to further identify the risk of bleeding and thrombosis, physicians could focus on leukocyte count and hs-CRP respectively for those in status of inflammation, providing new insights into individualized antiplatelet therapy.

## Data Availability Statement

The datasets presented in this article are not readily available due to the fact that ethical restrictions related to the consent given by subjects at the time of study commencement, and our datasets are available from the corresponding author upon reasonable request after permission of the Insti-tutional Review Board of State Key Laboratory of Cardiovascular Disease, Fuwai Hospital, National Center for Cardiovascular Diseases. Requests to access the datasets should be directed to Xueyan Zhao, zhao_xueyan@sina.com.

## Ethics Statement

The studies involving human participants were reviewed and approved by Fuwai hospital institutional review board. The patients/participants provided their written informed consent to participate in this study.

## Author Contributions

JL, XZ, and JY contributed to the conception or design of the work. JL, DY, LJ, XT, JX, YS, JC, SQ, and BX contributed to the acquisition, analysis, or interpretation of data for the work. JL contributed to statistical analysis and drafted the manuscript. XZ, YY, RG, and JY critically revised the manuscript. All authors gave final approval and agreed to be accountable for all aspects of work ensuring integrity and accuracy.

## Funding

This work was supported by the CAMS Innovation Fund for Medical Sciences (Grant number: 2020-I2M-C&T-B-052); National Key Research and Development Program of China (2016YFC1301300, 2016YFC1301301); Young and middle-aged talents in the XPCC Science and Technology Project (2020CB012); Key Science and Technology Project of Shihezi (2019ZH09).

## Conflict of Interest

The authors declare that the research was conducted in the absence of any commercial or financial relationships that could be construed as a potential conflict of interest.

## Publisher's Note

All claims expressed in this article are solely those of the authors and do not necessarily represent those of their affiliated organizations, or those of the publisher, the editors and the reviewers. Any product that may be evaluated in this article, or claim that may be made by its manufacturer, is not guaranteed or endorsed by the publisher.
