# Supplementary material for: Similar Inflammatory Biomarkers Reflect Different Platelet Reactivity in Percutaneous Coronary Intervention Patients Treated With Clopidogrel: A Large-Sample Study From China
Source: Front Cardiovasc Med. 2021 Oct 4;8:736466. doi: 10.3389/fcvm.2021.736466 (PMC8521006; doi:10.3389/fcvm.2021.736466)
Supplement: Supplementary file 1 [file Table_1.docx]

**Supplementary Table 1. Logistic regression for LTPR after propensity score matching**

| **Parameter** | **Univariate logistic regression** | | | **Multivariate logistic regression** | | |
| --- | --- | --- | --- | --- | --- | --- |
|  | **Crude OR** | **95% CI** | ***p* value** | **Adjusted OR** | **95% CI** | ***p* value** |
| Sex | 1.018 | 0.875-1.185 | 0.816 | - | - | **-** |
| Age | 1.000 | 0.994-1.005 | 0.808 | - | - | **-** |
| BMI | 0.974 | 0.956-0.991 | 0.004 | 0.973 | 0.955-0.991 | 0.003 |
| Leukocyte count | 1.067 | 1.035-1.100 | <0.001 | 1.128 | 1.091-1.166 | <0.001 |
| Hs-CRP | 0.942 | 0.926-0.958 | <0.001 | 0.923 | 0.906-0.939 | <0.001 |
| Smoking history | 0.977 | 0.870-1.098 | 0.699 | - | - | **-** |
| ACS | 0.917 | 0.818-1.028 | 0.137 | - | - | **-** |
| Hyperlipidemia | 1.047 | 0.927-1.182 | 0.458 | - | - | **-** |
| Hypertension | 0.952 | 0.846-1.072 | 0.416 | - | - | **-** |
| Diabetes mellitus | 1.006 | 0.888-1.140 | 0.924 | - | - | **-** |
| COPD | 1.227 | 0.825-1.835 | 0.313 | - | - | **-** |
| Family history of CHD |  |  |  | - | - | **-** |
| Cerebrovascular disease history | 0.986 | 0.814-1.193 | 0.884 | - | - | **-** |
| Peripheral vascular disease | 0.954 | 0.673-1.351 | 0.790 | - | - | **-** |
| Prior myocardial infarction | 1.042 | 0.906-1.199 | 0.567 | - | - | **-** |
| Prior PCI | 1.043 | 0.916-1.188 | 0.528 | - | - | **-** |
| Prior CABG | 0.922 | 0.697-1.219 | 0.569 | - | - | **-** |
| LVEF | 0.999 | 0.991-1.007 | 0.803 | - | - | **-** |
| Hemoglobin | 0.999 | 0.995-1.003 | 0.693 | - | - | **-** |
| PLT | 1.000 | 1.000-1.001 | 0.411 | - | - | **-** |
| MPV | 0.997 | 0.938-1.060 | 0.931 | - | - | **-** |
| LDL-C | 0.973 | 0.912-1.039 | 0.419 | - | - | **-** |
| HDL-C | 1.217 | 0.981-1.511 | 0.075 | - | - | **-** |
| TC | 0.980 | 0、927-1.035 | 0.464 | - | - | **-** |
| Triglyceride | 1.006 | 0.953-1.062 | 0.824 | - | - | **-** |
| Glucose | 1.004 | 0.973-1.036 | 0.793 | - | - | **-** |
| eGFR | 1.001 | 0.997-1.005 | 0.708 | - | - | **-** |

BMI, body mass index; MA(ADP), adenosine diphosphate (ADP)–induced platelet maximum amplitude; WBC, white blood cell; Hs-CRP, high-sensitivity C reactive protein; ACS, acute coronary syndrome; COPD, chronic obstructive pulmonary disease; CHD, coronary heart disease; PCI, percutaneous coronary intervention; CABG, coronary artery bypass graft; LVEF, left ventricle ejection fraction; PLT, platelet count; MPV, mean platelet volume; LDL-C, low-density lipoprotein cholesterol; HDL-C, high-density lipoprotein cholesterol; TC, total cholesterol; eGFR, estimated glomerular filtration rate
