# Supplementary material for: Similar Inflammatory Biomarkers Reflect Different Platelet Reactivity in Percutaneous Coronary Intervention Patients Treated With Clopidogrel: A Large-Sample Study From China
Source: Front Cardiovasc Med. 2021 Oct 4;8:736466. doi: 10.3389/fcvm.2021.736466 (PMC8521006; doi:10.3389/fcvm.2021.736466)
Supplement: Supplementary file 2 [file Table_2.docx]

**Supplementary Table 2. Logistic regression for HTPR after propensity score matching**

| **Parameter** | **Univariate logistic regression** | | | **Multivariate logistic regression** | | |
| --- | --- | --- | --- | --- | --- | --- |
|  | **Crude OR** | **95% CI** | ***p* value** | **Adjusted OR** | **95% CI** | ***p* value** |
| Sex | 0.986 | 0.860-1.130 | 0.835 | - | - | **-** |
| Age | 1.001 | 0.994-1.007 | 0.865 | - | - | **-** |
| BMI | 1.038 | 1.017-1.059 | <0.001 | 1.036 | 1.015-1.090 | 0.098 |
| Leukocyte count | 0.960 | 0.928-0.992 | 0.014 | 0.900 | 0.866-0.931 | <0.001 |
| Hs-CRP | 1.072 | 1.054-1.091 | <0.001 | 1.091 | 1.071-1.111 | <0.001 |
| Smoking history | 1.009 | 0.888-1.146 | 0.897 | - | - | **-** |
| ACS | 1.029 | 0.904-1.171 | 0.667 | - | - | **-** |
| Hyperlipidemia | 0.978 | 0.853-1.122 | 0.753 | - | - | **-** |
| Hypertension | 1.012 | 0.884-1.158 | 0.864 | - | - | **-** |
| Diabetes mellitus | 1.005 | 0.877-1.152 | 0.945 | - | - | **-** |
| COPD | 0.845 | 0.563-1.263 | 0.413 | - | - | **-** |
| Family history of CHD |  |  |  | - | - | **-** |
| Cerebrovascular disease history | 1.005 | 0.822-1.229 | 0.959 | - | - | **-** |
| Peripheral vascular disease | 1.44 | 0.990-2.115 | 0.058 | - | - | **-** |
| Prior myocardial infarction | 0.935 | 0.792-1.103 | 0.423 | - | - | **-** |
| Prior PCI | 0.948 | 0.814-1.103 | 0.487 | - | - | **-** |
| Prior CABG | 0.859 | 0.619-1.190 | 0.361 | - | - | **-** |
| LVEF | 1.001 | 0.992-1.001 | 0.847 | - | - | **-** |
| Hemoglobin | 1.000 | 1.000-1.004 | 0.960 | - | - | **-** |
| PLT | 1.000 | 1.000-1.001 | 0.956 | - | - | **-** |
| MPV | 1.112 | 1.036-1.193 | 0.003 | - | - | **-** |
| LDL-C | 1.000 | 0.931-1.073 | 0.984 | - | - | **-** |
| HDL-C | 0.800 | 0.630-1.003 | 0.053 | - | - | **-** |
| TC | 1.007 | 0.949-1.070 | 0.811 | - | - | **-** |
| Triglyceride | 1.038 | 0.973-1.107 | 0.259 | - | - | **-** |
| Glucose | 0.989 | 0.959-1.020 | 0.497 | - | - | **-** |
| eGFR | 0.999 | 0.995-1.003 | 0.694 | - | - | **-** |

BMI, body mass index; MA(ADP), adenosine diphosphate (ADP)–induced platelet maximum amplitude; WBC, white blood cell; Hs-CRP, high-sensitivity C reactive protein; ACS, acute coronary syndrome; COPD, chronic obstructive pulmonary disease; CHD, coronary heart disease; PCI, percutaneous coronary intervention; CABG, coronary artery bypass graft; LVEF, left ventricle ejection fraction; PLT, platelet count; MPV, mean platelet volume; LDL-C, low-density lipoprotein cholesterol; HDL-C, high-density lipoprotein cholesterol; TC, total cholesterol; eGFR, estimated glomerular filtration rate
